# Validation of the food frequency questionnaire for the assessment of dietary vitamin D intake

**DOI:** 10.3389/fnut.2022.950874

**Published:** 2022-09-23

**Authors:** Maša Hribar, Katarina Žlavs, Igor Pravst, Katja Žmitek

**Affiliations:** ^1^Nutrition and Public Health Research Group, Nutrition Institute, Ljubljana, Slovenia; ^2^Biotechnical Faculty, University of Ljubljana, Ljubljana, Slovenia; ^3^VIST–Faculty of Applied Sciences, Ljubljana, Slovenia

**Keywords:** vitamin D, nutrient intake, validation, reproducibility, Slovenia, Food Frequency Questionnaire (FFQ), dietary record

## Abstract

Vitamin D and its adequate status are related to many aspects of human health; therefore, an appropriate tool is needed for the valid assessment of vitamin D status. The main contributor to vitamin D status is endogenous synthesis after cutaneous exposure to ultraviolet B light (UVB), but in the absence of UVB radiation, vitamin D intake becomes an important source of vitamin D. Various tools are available for vitamin D intake assessments, with the Food Frequency Questionnaire (FFQ) being among the fastest, cheapest, and most convenient; however, until now, these tools have not been adapted for the Slovenia (SI). To enable valid vitamin D intake estimation, we developed a simple one-page semi-quantitative FFQ (sqFFQ/SI) and tested its validity using a 5-day dietary record (DR) as a reference method. The reproducibility was tested with the second sqFFQ/SI (sqFFQ/SI2) 6 weeks after the first (sqFFQ/SI1). The validity and reproducibility of this method were tested on 54 participants using Bland–Altman plots, Spearman’s correlation, and Kappa analyses of tertiles. The mean daily vitamin D intake was 3.50 ± 1.91 μg according to the 5-day DR, and 2.99 ± 1.35 and 3.31 ± 1.67 μg according to the sqFFQ/SI1 and repeated sqFFQ/SI (sqFFQ/SI2), respectively. When analyzing for validity, the sqFFQ/SI1 was found to be significantly correlated (*p* < 0.05) with the 5-day DR, with an acceptable correlation coefficient of 0.268 and a Bland–Altman index of 3.7%. For reproducibility, the correlation between the sqFFQ/SI1 and sqFFQ/SI2 was highly significant (*p* < 0.001), with a good correlation coefficient of 0.689 and a Bland–Altman index of 3.7%. Kappa analyses of tertiles showed a poor validity and acceptable reproducibility. Overall, we observed a higher reproducibility than validity. Validation and reproducibility analyses demonstrated that the proposed sqFFQ/SI is acceptable and is, therefore, an appropriate tool for the effective assessment of habitual vitamin D intake on an individual level. With this consideration, this tool will be used in further population studies to assess vitamin D intake and for the development of a screening tool for the assessment of the risk for vitamin D deficiency, which will be used as a foundation for evidence-based policy-making decisions.

## Introduction

Vitamin D is a fat-soluble vitamin that is, due to its many functions in the body, crucial for the growth and maintenance of health in all life stages (1–3). For humans, the sources of vitamin D are endogenous synthesis in the skin when exposed to ultraviolet B (UVB) radiation and dietary intake (either with foods that are naturally rich in vitamin D, fortified foods, or pharmaceutical preparations) (4). Although endogenous synthesis is the main source of vitamin D for most people, in the absence of sufficient UVB exposure, vitamin D becomes an essential nutrient and sufficient dietary intake is required (5–7).

The dietary vitamin D intake is usually well below recommendations (5), mainly because very few foods are rich in vitamin D, and, at the same time, they are seldom consumed. The recommended dietary vitamin D intake for the adult population is 5 μg/day (19–50 years), 10 μg/day (51–65 years), and 15 μg/day (>65 years) according to the recommendations of the World Health Organization (WHO) (8), and 15 and 20 μg/day (in the absence of endogenous synthesis) according to the recommendations of the European Food Safety Authority (EFSA) and the Nutrition Societies of Germany, Austria, and Switzerland (D-A-CH), respectively (9, 10). On the other hand, the nutrient reference value (NRV), as defined in the European union food labeling regulation, is 5 μg (11), while the threshold of 2.5 μg is sometimes used as a lower reference nutrient intake (LRNI) (12).

Studies have reported a high prevalence of inadequate dietary intakes of vitamin D in European populations and around the world (6, 13, 14), including Slovenia (15, 16). In most European countries, the daily intake of vitamin D is lower than 5 μg (6, 14, 16, 17); exceptions are Scandinavian countries, where oil-rich fish consumption is relatively high, and both fortification and supplementation policies have also been implemented (6, 18). Only a few studies have evaluated the dietary intake of vitamin D in the Slovenian population, using various methods to record the dietary intake (15, 16).

Due to insufficient UVB exposure and simultaneous inadequate vitamin D intake, an important public health task is the rapid identification of individuals exposed to the risk of inadequate vitamin D status. Furthermore, the accurate assessment of dietary vitamin D intake is important for the application of evidence-based public health measures in order to prevent poor vitamin D status in different population groups. To achieve this, a suitable screening procedure is necessary (19).

The optimal and most objective method for evaluating vitamin D status is a laboratory determination of serum 25-hydroxyvitamin D [25(OH)D] (20), but this method is invasive and not recommended for screening in large populations (19, 21). Because vitamin D status is also affected by dietary intake of vitamin D, a valid dietary assessment method that is easy to use is needed (22). Determining dietary vitamin D intake with the 24-h recall or the dietary record (DR) method, a gold standard for dietary intake assessments, is not the most well suited (23); because of large day-to-day variations in vitamin D intake (dependent on, e.g., fish intake and the diversity of fortified foods), an extended time period is necessary for data collection (22, 24). On the other hand, the Food Frequency Questionnaire (FFQ) is less useful for measuring the absolute dietary intake, but it can better reflect one’s typical diet (25). Additionally, the FFQ may be more reliable for the estimation of micronutrient intake, such as vitamin D, as it covers a longer period and can focus on specific foods that are relevant to vitamin D intake (26).

When assessing dietary intake, the research method must be simple and fast, for both the subject and the researcher, and, at the same time, it must be valid and reproducible (25, 27). Various FFQs for the assessment of vitamin D intake were designed and validated in several countries and studies around the world (23, 26, 28–34). However, such tools need to be tailored for use in specific regions; country-specific food consumption patterns and foods need to be considered (25, 27, 35). The typical reference methods for the validation of the FFQ are DR or the 24-h recall method, and biomarkers are sometimes also used (25).

The objective of this study was to assess the validity and reproducibility of a semi-quantitative FFQ on the Slovenian population (sqFFQ/SI) for the assessment of the dietary intake of vitamin D, using 5-day DR as a reference method. The sqFFQ/SI was developed by the Nutrition Institute (Slovenia) in cooperation with the National Institute of Public Health (Slovenia) within the national research project Nutri-D “Challenges in achieving adequate vitamin D status in the adult population” (L7-1849).

## Materials and methods

### Study design and data collection

**FIGURE 1:**
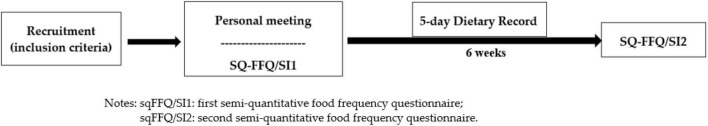
Study design.

The study protocol was approved by the Nutrition Research Ethics Committee (Biotechnical Faculty, University of Ljubljana), under the identification number KEP-1-2/2020 on 10 February 2020. The study was conducted in full compliance with the principles laid out in the Declaration of Helsinki. Participation in the study was voluntary. All of the subjects signed a written informed consent form before participating. They were informed that they can withdraw from the study at any time with no consequences. The study was conducted between February and April 2020 and included data collection using the FFQ and a 5-day DR. The participants received all the required information (and instructions) in oral and written format at individual meetings. To assess reproducibility, the participants were asked to fill out the sqFFQ/SI two times: the first one (sqFFQ/SI1) was filled out at the beginning of the study, and the second one (sqFFQ/SI2) was filled out approximately 6 weeks later (Figure 1). The participants were asked not to alter dietary habits between sqFFQ/SI1 and sqFFQ/SI2 if possible. It should be noted that the second one was conducted during the SARS-CoV-2 epidemic. To evaluate the validity of the questionnaire, the participants were requested to complete a 5-day DR during the time between both administered sqFFQ/SI. The participants were free to choose any 3 week/2 weekend days in that time period.

### Study population

The sqFFQ/SI was validated among a group of Slovenian adults, aged between 18 and 65 years, mainly from central Slovenia and the Savinja statistical region. The subjects were enrolled with the use of invitations *via* social media profiles from the official Nutrition Institute profile, and personal invitations. The exclusion criteria were diagnosis of chronic disease, pregnant or breastfeeding women, and specific diets (vegan diet, ketogenic diet, energy-restricted diets, and diets due to medical reasons). It should be noted that vegetarians were not excluded. All the required information regarding inclusion/exclusion criteria was presented before the beginning of the study.

### Semi quantitative food frequency questionnaire for Slovenian population

For this study, a semi-quantitative FFQ adapted for the Slovenian population was used (sqFFQ/SI), in which the frequency of food consumption and the size of portions are defined (36). The tool included food products that were previously identified as important sources of vitamin D in Slovenia (15). Although Slovenia does not have a mandatory vitamin D fortification of foods, some food groups are commonly fortified (37) and were therefore included. The final sqFFQ/SI consisted of 22 food items that contain at least 0.03 μg of vitamin D per 100 g, according to the reviewed literature (38) and the selected food composition databases: Slovenian Open Platform for Clinical Nutrition (OPEN) (39), McCance and Widdowson’s The Composition of Foods (38), and the United States Department of Agriculture (USDA) database (40). The included food groups are presented in Table 1. For each food group, we identified all the relevant food records in the abovementioned food composition datasets and calculated the category average content of vitamin D. We did not include the use of pharmaceutical preparations.

**TABLE 1:** Reference serving sizes and vitamin D content in 100 g of the foods used in the semi-quantitative Food Frequency Questionnaire (sqFFQ/SI).

Food group	Reference serving size (g/ml)	Vitamin D (μg/100 g)
Sardines, trout, salmon, and carp	120	7.84
Sea bass, tuna, cod, common sole, blue tilapia, and other fish	120	3.23
Canned fish	80	4.31
Plant-based milk alternatives: rice milk, soy milk, etc.	250	0.47
Semi-skimmed milk (1.5% milkfat), cocoa drink, and milk drinks	200	0.03
Whole milk (3.5% milkfat), a cocoa drink containing whole milk, milk drinks	200	0.09
Semi-skimmed (1.5% milkfat) flavored or plain yogurt	150	0.03
Whole milk (3.5% milkfat) flavored or plain yogurt	150	0.06
Hard cheese: Gouda cheese, Edam cheese, etc.	30	0.9
Blue cheese	20	0.39
Cottage cheese, mozzarella, other types of processed cheese	50	0.28
Ice cream	40	0.25
Butter	6	1.66
Margarine	6	2.5
Eggs	50	2.9
Egg pasta	100	0.28
Red meat	100	0.48
Poultry	100	0.26
Meat products	40	0.86
Calf’s liver	60	1.2
Mushrooms	100	0.18
Cakes, pastry, and muffins	70	0.31

The subjects were asked to rank their consumption frequencies during the past year. Previously reported (17) frequency options were implemented: multiple times a day, daily, 4–6 times per week, 1–3 times per week, 1–3 times per month, and rarely or never. Further, subjects were asked to rank their usual portion sizes (in comparison to the indicated reference portion size): (a) as indicated, (b) less than indicated (specified as at least one-half smaller than the normal portion size), and (c) more than indicated (specified as at least one-half larger than the normal portion size). The complete sqFFQ/SI is provided in the Supplementary material. The sqFFQ/SI was carried out online and took approximately 10 min to complete.

### Five-day dietary record

In line with the previously reported approach (31), the 5-day DR was conducted on five typical random non-consecutive days (3 weekdays and 3 days during the weekend). At the first meeting, the participants were given detailed instructions on how to complete the DR. Participants were asked to maintain their usual eating habits and record all consumed foods and beverages in as much detail as possible (describing the type/brand of food, the amount of food, the method of preparation, and the recipes of composited dishes where applicable). The amounts were preferably weighted and written down in grams when participants had access to a kitchen scale. Exceptionally, the amounts were estimated using illustration material for different portion sizes of typical foods using a previously developed nationally adapted picture book (41). The participants returned their completed 5-day DR *via* a pre-paid postal service or in person.

### Data processing and statistical analysis

The data collected by both sqFFQ/SI were used to calculate the daily vitamin D intake (μg/day) based on the method described in detail by Biro and Gee (42). The calculations were performed using the selected serving size and average vitamin D contents in 100 g of foods, as shown in Table 1.

Vitamin D intake (μg/day) was further determined using a 5-day DR using the online nutrition analysis software OPEN, which is linked to the food composition database (43). Due to some missing information regarding the vitamin D content in some foods, the OPEN database was updated in cooperation with the software owner, the Jožef Stefan Institute (JSI). The missing data were updated with data available in the USDA database (40), the National Food Composition Database in Finland (Fineli) (44), and McCance and Widdowson’s The Composition of Foods (38).

The obtained data were statistically analyzed with the IBM SPSS version 27, Statistics program (IBM SPSS, IBM Corp., Armonk, NY, USA) (45). We investigated the validity (external validation compared with the results of the 5-day DR) and reproducibility of the method (internal validation comparing results obtained two times: sqFFQ/SI1 and sqFFQ/SI2) (46). Descriptive characteristics (means, median, and proportions) for the daily vitamin D intakes were calculated.

The estimated daily vitamin D intakes were grouped for cross-classification according to tertiles. In the analyses, we regarded the estimations as good if less than 10% of the participants were grossly misclassified into the opposite tertiles and at least 50% of the participants were correctly classified (47). In the Kappa analyses, we considered Kappa values below 0.20 to have a poor agreement, between 0.20 and 0.60 as having an acceptable agreement, and over 0.60 as having good agreement (48).

The normality of distribution was tested with the Shapiro–Wilk test. The analysis of correlations between the results obtained in the assessment of validity (sqFFQ/SI1 compared with a 5-day DR) and the assessment of reproducibility (comparison between sqFFQ/SI1 and sqFFQ/SI2) was used, where Spearman’s correlation was applied. Correlation coefficients of less than 0.20 were a poor outcome; those between 0.20 and 0.49 were acceptable, and those of 0.50 or higher was considered a good outcome (48).

In all of the comparisons, significance was considered at *p* < 0.05. A Bland–Altman plot was further used for the validation and reproducibility assessment. Since the data were not normally distributed, we used log transformation, as previously proposed (49). A Bland–Altman index below 5% was interpreted as good, as suggested before in similar research (23, 28, 49–51).

## Results

A total of 55 participants volunteered to participate. The final sample included 54 participants, as one of the individuals withdrew from the study (due to lack of time). The sample was represented by 37 women (69%) and 17 men (31%). The average age was 32.7 years (±13.6 years). Other characteristics of the population [age and body mass index (BMI)] are shown in Table 2. The participants completed two sqFFQ/SIs on average 46 days apart (sqFFQ/SI1 and sqFFQ/SI2, respectively), and a 5-day DR according to a study design, presented in Figure 1. The mean daily vitamin D intake was 3.50 ± 1.91 μg according to the 5-day DR, and 2.99 ± 1.35 and 3.31 ± 1.67 μg according to the sqFFQ/SI1 and sqFFQ/SI2, respectively (Table 3). Since none of the participants achieved the nationally recommended daily intake of vitamin D (20 μg), we analyzed the data with a cut-off value for the LRNI (2.5 μg) and NRV (5 μg). Overall, the NRV threshold was not met by 87.0% of subjects according to the 5-day DRs, 90.7% according to the sqFFQ/SI1, and 83.3% according to the sqFFQ/SI2. On the other hand, the lower LRNI threshold was not met by 35.2, 42.6, and 40.7%, respectively.

**TABLE 2:** Study population description.

Parameter	Criteria	Number (%)
Participants (total)		54 (100)
Sex	Men	17 (31.5)
	Women	37 (69.5)
Age	19–24	28 (52.9)
	25–65	26 (48.1)
Body mass index categories	<18.5 kg/m^2^ (underweight)	2 (3.7)
	18.5–24.9 kg/m^2^ (normal weight)	37 (68.5)
	25.0–29.9 kg/m^2^ (overweight)	10 (18.5)
	>30.0 kg/m^2^ (obese)	5 (9.3)

**TABLE 3:** Daily vitamin D intake estimated with a 5-day dietary record (DR) and semi-quantitative Food Frequency Questionnaires (sqFFQ/SI) administered 6 weeks apart.

	sqFFQ/SI1	sqFFQ/SI2	5-day DR
Mean ± SD (μg)	2.99 ± 1.35	3.31 ± 1.67	3.50 ± 1.91
Median (μg)	2.61	2.94	3.04
Minimum (μg)	0.44	0.58	0.97
Maximum (μg)	7.08	8.19	10.31
<2.5 μg (%)	42.6	40.7	35.2
<5 μg (%)	90.7	83.3	87

### Validity

**FIGURE 2:**
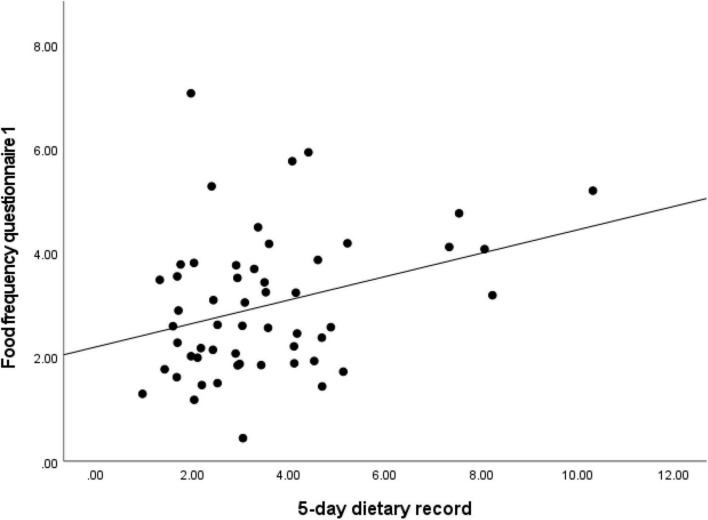
Analysis of correlation for daily vitamin D intake estimated with semi-quantitative Food Frequency Questionnaire 1 (sqFFQ/SI1) and 5-day dietary record (correlation coefficient = 0.268; *p* < 0.05).

**FIGURE 3:**
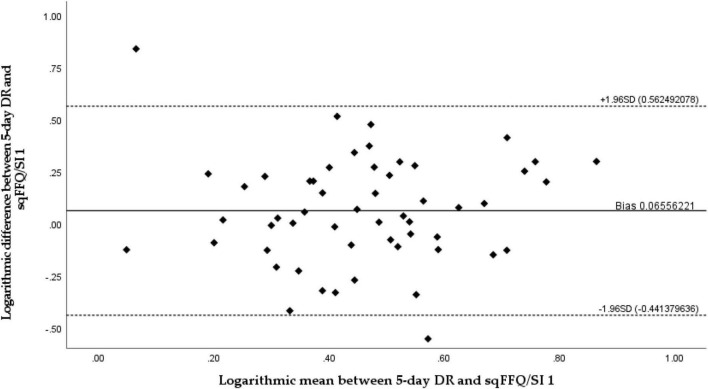
Bland–Altman plot comparing daily vitamin D intake estimated with semi-quantitative Food Frequency Questionnaire 1 (sqFFQ/SI1) and a 5-day dietary record (Bland–Altman index: 3.70%).

The validity of the sqFFQ/SI1 for the estimation of daily vitamin D intake was analyzed with comparison to the 5-day DR. The estimated intakes were analyzed with Spearman’s rank correlation for the sqFFQ/SI1 and 5-day DR (Figure 2). The sqFFQ/SI1 was significantly correlated (*p* < 0.05) with the 5-day DR, with a correlation coefficient of 0.268; the mean difference between both methods was 0.514 μg (SD: 0.318 μg). Due to the non-normal distribution, further comparison of the 5-day DR and sqFFQ/SI1 using Bland–Altman plots were carried out with log-transformed data (Figure 3). The Bland–Altman index of the logarithmic model was good (3.70%). Further, we analyzed the percentages of subjects classified into the same vitamin D intake tertile (Table 4). When comparing the sqFFQ/SI1 and the 5-day DR, 42.6% of the participants were categorized into the same tertile, and 16.7% into the opposite tertile. This indicates a low agreement; the Kappa coefficient was 0.139.

**TABLE 4:** Count and percentages of subjects classified into the same/opposite vitamin D intake tertile.

Category		sqFFQ/SI1 vs. 5-day DR	sqFFQ/SI1 vs. sqFFQ/SI2
Subjects classified into the same tertile	*N*	23	32
	%	42.6	59.3
Subjects misclassified into the opposite tertile	*N*	9	0
	%	16.7	0

DR, dietary record; sqFFQ/SI1, semi-quantitative Food Frequency Questionnaire 1; sqFFQ/SI2, semi-quantitative Food Frequency Questionnaire 2.

### Reproducibility

**FIGURE 4:**
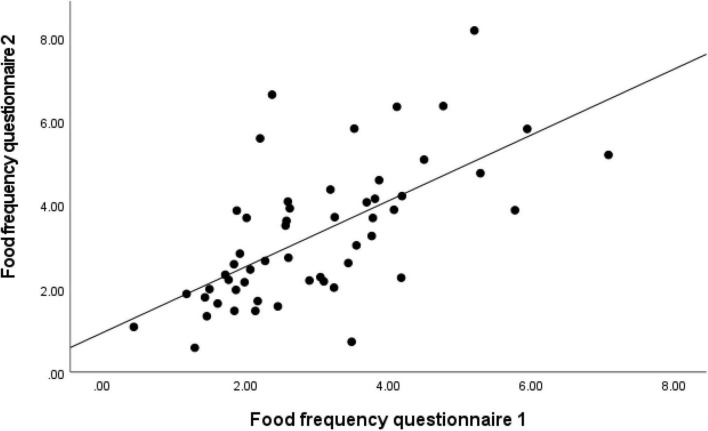
Analysis of correlation for daily vitamin D intake estimated with semi-quantitative Food Frequency Questionnaire 1 (sqFFQ/SI1) and 2 (sqFFQ/SI2) (correlation coefficient = 0.689; *p* < 0.001).

**FIGURE 5:**
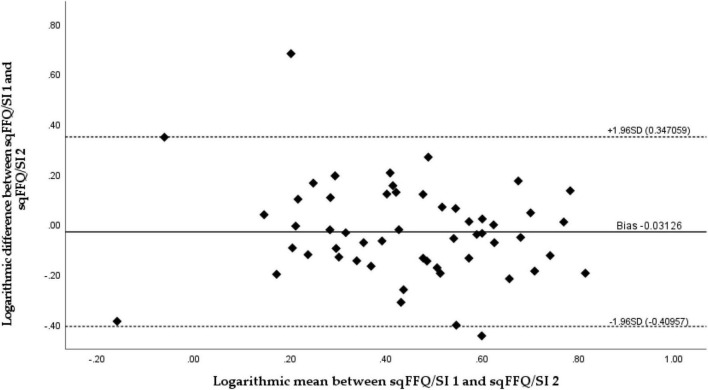
Bland–Altman plot comparing daily vitamin D intake estimated with a semi-quantitative Food Frequency Questionnaire 1 (sqFFQ/SI1) and 2 (sqFFQ/SI2) (Bland–Altman index: 3.70%).

To investigate the reproducibility of the sqFFQ/SI, we compared the daily vitamin D intake as estimated with the sqFFQ/SI1 and sqFFQ/SI2, which were administered approximately 6 weeks apart. The correlation between the sqFFQ/SI1 and sqFFQ/SI2 was highly significant (*p* < 0.001), with a correlation coefficient of 0.689 (Figure 4). The mean difference between measurements was 0.318 μg (SD: 0.291 μg). Furthermore, the log-transformed Bland–Altman plot showed good reproducibility with an index of 3.70% (Figure 5). When testing the sqFFQ/SI1 for reproducibility, the analysis of the tertiles showed acceptable agreement; 59.3% of the subjects were categorized into the same tertile, and there were no classifications into opposite tertile, while the Kappa value was acceptable (0.389) (Table 4).

## Discussion

Vitamin D is a crucial micronutrient for optimal human health in all life stages, and we should strive to achieve optimal status across all populations. Besides UVB-induced cutaneous synthesis, food intake is an important source of vitamin D (5–7). Vitamin D intake can be estimated using various methods, with the FFQ being one of the less burdensome methods. The FFQ is user friendly and time/cost efficient (25). Convenient tools for intake estimation are important for the efficient assessment of the risk of vitamin D deficiency, particularly in the absence of endogenous synthesis. To accurately assess the dietary intake of vitamin D in the Slovenian population we developed a semi-quantitative FFQ and tested its validity and reproducibility using 5-day DR and repeated sqFFQ/SI, respectively. The estimated mean daily vitamin D intakes in our study were 3.50, 2.99, and 3.31 μg for the 5-day DR, sqFFQ/SI1, and sqFFQ/SI2, respectively. We did not observe a higher mean intake with the FFQ (in comparison to the 5-day DR), unlike some other validation studies (29, 52).

The validity and reproducibility were tested using Bland–Altman plots, a recommended “gold-standard” approach by which to compare results from different methods observing the same variable (53). Our results show that the developed sqFFQ/SI is fairly valid and reproducible; only 3.70% of the data points were outside the 95% limits of agreements for both validity and reproducibility. Other research investigating similar a topic reported from 2.7 to 6.3% of data points outside the 95% limits of agreement using Bland–Altman plot (23, 28). Additionally, Spearman’s correlation was significant both for validity (<0.05) and reproducibility (<0.001). The correlation coefficients were acceptable and good (0.268 and 0.689, respectively). In similar studies comparing multiple day dietary vitamin D intake with FFQ, significant correlation coefficients ranged from 0.21 to 0.83 for validity and from 0.62 to 0.82 for reproducibility (23, 26, 28, 29, 52). It should be noted that due to the complexity of the estimation of micronutrient intakes, correlation coefficients above 0.2 are considered acceptable, and coefficients above 0.7 are rarely reported (32). However, the thresholds for acceptable correlations are not well harmonized (25, 48), and we should take caution when evaluating the outcomes. Analyses of terciles in our case showed less agreement than in some other studies. Altogether, in the validity study, 42.6% of subjects were classified in the same tercile (Kappa coefficient: 0.139; poor agreement), while some other studies reported up to 64% (28, 29), but we must note that the cross-classification is a relatively crude measurement (29). On the hand, we observed better results in analyses of terciles in the reproducibility study (59.3%; Kappa coefficient: 0.389; acceptable). Other studies also reported lower differences in the reproducibility of FFQs, in comparison to validity testing with DRs (23, 54), which might be affected by the limited ability of DRs to capture dietary patterns, related to vitamin D intake.

In a recent study, it was shown that in Slovenia vitamin D deficiency is highly prevalent, particularly in the wintertime when dietary intake becomes the main source of vitamin D. In the winter months, ca. 80% of adults and elderly people were shown to be vitamin D deficient (55), and the mean daily vitamin D intakes were 2.9 and 2.5 μg, respectively (15). Globally, various FFQs were developed and regionally adapted to estimate vitamin D intakes (23, 26, 28–34); however, to the best of our knowledge, there is no such tool available for use in the Slovenian population.

The intake of nutrients can be estimated using a variety of methods that have different levels of accuracy for different nutrients. For nutrients that are found in a limited number of foods, the use of short-period DRs can pose a risk of not capturing a typical dietary pattern, and it is therefore recommended to follow food intake over a period of several days. On the contrary, although the FFQ is much simpler to use, this method can better capture food consumption patterns over a longer period (26). In the case of vitamin D, the intake estimation is particularly challenging due to notable day-to-day variations as vitamin D-rich foods (i.e., fish) are seldom consumed (24, 26). This, of course, affects the estimation of daily vitamin D intake when different methods are used. For example, in a nationally representative Slovenian SI. Menu study, 72.8% of adults were recognized as sea fish consumers when two 24 h dietary recalls were used, while the Food Propensity Questionnaire method identified 80.8% as true consumers (15). We developed an FFQ that covers the most important contributors to vitamin D intake in Slovenia, including eggs, fish, and fish products, meat and meat products, milk and milk products, and commonly fortified foods, such as plant-based milk alternatives (15, 37). The validation of the FFQ (sqFFQ/SI1) was conducted on 54 participants using a 5-day DR as a reference method. Despite the abovementioned limitations, the DR is a commonly used reference method in such validation studies (56). We also tested the reproducibility, using a repeated FFQ (sqFFQ/SI2) administered 6 weeks after the first measurement.

To evaluate the validity and reproducibility we used various approaches. The results are showing that validity varied from poor to good, and good for reproducibility. We have demonstrated that the proposed FFQ is acceptable and is therefore an appropriate tool for the effective assessment of habitual vitamin D intake on an individual level. Overall, we observed higher reproducibility than validity. However, such tools are also commonly used in population studies. Therefore, we further compared the estimated mean vitamin D intakes between the tested methods and literature data. The difference between the mean vitamin D intake according to both of the tested methods was small (0.51 μg) and statistically insignificant. With consideration of the recommended daily vitamin D intake (20 μg), the clinical importance of such a difference is minimal. Similar differences were also observed in other similar studies, for example, in the study by Kiely et al. in their comparison of the FFQ and 14-day DR results (29). Furthermore, our results are comparable with mean vitamin D intakes reported for the general Slovenian population. Vitamin D intake was recently investigated in a nationally representative SI. Menu study (15). The weighted population mean intake was estimated with the multiple source method (MSM), using two 24 h recalls and the Food Propensity Questionnaire. The estimated mean vitamin D intake in adults (18–64 years) was 2.85 μg (15), comparable to the results in our study (sqFFQ/SI1: 2.99 μg). A recent systematic review also highlighted that vitamin D intakes in other studies in the Slovenian population were below 5 μg (16).

Although the developed tool was shown as valid and reproducible, some limitations need to be noted. While we followed the recommendation that validity studies should be conducted on at least 50 subjects (31), a bigger sample would be beneficial to check the validity in more specific population groups. Furthermore, we did not use biological biomarkers of vitamin D status [serum 25(OH)D concentration], but we should note that this biomarker is seriously affected by UVB-induced endogenous vitamin D biosynthesis, which results in major inter-individual differences. The limited use of blood biomarkers for such validation studies in the case of vitamin D was noted also in other studies (26, 33). Moreover, we should note that while majority (72.2%) of our study participants were with BMI < 25 kg/m^2^, we also had some overweight/obese subjects (18.5 and 9.3%, respectively), where food intake misreporting might be more common. We have not excluded those from the analyses, because vitamin D intake screening is also very relevant in this population group. At last, it should be said that we tested the tool on healthy, non-pregnant, no-lactating, adult, omnivore populations. We suggest that the described sqFFQ/SI is further tested on other populations of public health interest.

## Conclusion

The estimation of one’s usual daily vitamin D intake is a challenging task, regardless of the method used, due to its major day-to-day variability. Building on previously established methods and major contributors to vitamin D intake in the Slovenian population, we developed a simple one-page semi-quantitative FFQ (sqFFQ/SI) for the quick estimation of one’s usual daily vitamin D intake. To the best of our knowledge, the described tool is the first FFQ adapted for the Slovenian population. The Bland–Altman plot analyses showed a good level of agreement between the developed sqFFQ/SI and the standard 5-day DR method, as well as a good reproducibility, with less than 5% of the outliers falling outside of the agreement limit and a significant correlation being observed. Further analyses of correlation showed acceptable and good correlation, whereas Kappa analyses of terciles showed poor and acceptable agreement tor validity and reproducibility, respectively. Considering the analyses results, this tool will be used in further population studies and for the development of a screening tool for the assessment of the risk for vitamin D deficiency in healthy non-pregnant, no-lactating, adult, and omnivore populations. Due to the high prevalence of vitamin D deficiency, such a method is important not only for researchers but also for clinical practice and policymakers. It should be noted that the developed tool is very valuable for use in other countries in the Central European region due to similar food policies and dietary patterns. However, minor modifications might be appropriate for specific populations.

## Data availability statement

The raw data supporting the conclusions of this article will be made available by the authors, without undue reservation.

## Ethics statement

The studies involving human participants were reviewed and approved by the Nutrition Research Ethics Committee (Biotechnical Faculty, University of Ljubljana), KEP-1-2/2020. The patients/participants provided their written informed consent to participate in this study.

## Author contributions

KŽm and IP: conceptualization, funding acquisition, methodology, and supervision. MH and KŽl: data curation, formal analysis, investigation, and writing—original draft. KŽm: project administration and resources. MH: validation and visualization. MH, KŽm, KŽl, and IP: writing—review and editing. All authors contributed to the article and approved the submitted version.
