# Supplementary material for: Validation of the food frequency questionnaire for the assessment of dietary vitamin D intake
Source: Front Nutr. 2022 Sep 23;9:950874. doi: 10.3389/fnut.2022.950874 (PMC9537601; doi:10.3389/fnut.2022.950874)
Supplement: Supplementary file 1 [file Table_1.DOCX]

**Supplementary Material**: Semi-quantitative food frequency questionnaire for estimation of vitamin D intake (sqFFQ/SI)

**We would like to know how frequently** you have consumed some foods **in the previous year** (on average). For each food group a size of normal portion in parenthesis is written. If the size of the portion that you have consumed is similar to the portion written in parentheses, please mark »as indicated«. If you normally consume a smaller portion (half smaller than the normal one), please mark »less than indicated« or if you consume a larger portion (larger for at least half of the normal one), please mark »more than indicated« (you should mark frequency as well as portion size, so two circles).

|  | Multiple daily | Daily | Weekly  4-6x | Weekly  1-3x | Monthly 1-3x | Rarely or never | Less than indicated | As indicated | More than indicated | Never |
| --- | --- | --- | --- | --- | --- | --- | --- | --- | --- | --- |
| Sardines, trout, salmon, carp (1 fillet, 120 g) | 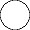 | 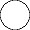 | 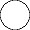 | 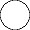 | 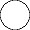 | 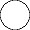 | 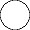 | 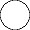 | 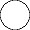 | 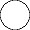 |
| Sea bass, tuna, cod, common sole, blue tilapia and other fish (1 fillet, 120 g) | 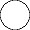 | 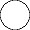 | 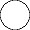 | 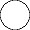 | 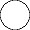 | 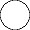 | 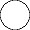 | 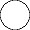 | 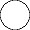 | 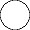 |
| Canned fish (1 can, 80 g) | 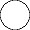 | 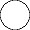 | 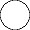 | 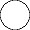 | 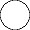 | 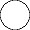 | 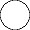 | 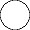 | 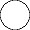 | 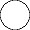 |
| Milk substitutes – rice milk, soy milk etc. (1 glass, 250 ml) | 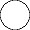 | 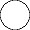 | 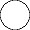 | 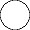 | 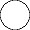 | 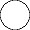 | 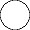 | 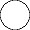 | 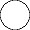 | 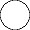 |
| Semi-skimmed milk (1.5% milk fat), cocoa drink, milk drinks  (1 glass, 200 ml) | 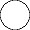 | 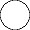 | 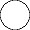 | 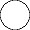 | 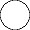 | 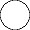 | 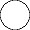 | 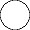 | 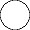 | 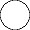 |
| Whole milk (3.5% milk fat), cocoa drink containing whole milk, milk drinks (1 glass, 200 ml) | 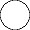 | 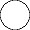 | 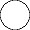 | 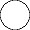 | 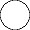 | 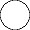 | 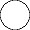 | 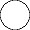 | 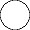 | 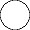 |
| Semi-skimmed (1.5% milk fat) flavoured or plain yogurt (1 pot, 150 g) | 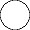 | 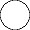 | 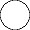 | 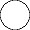 | 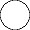 | 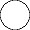 | 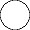 | 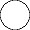 | 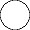 | 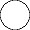 |
| Whole milk (3.5% milk fat) flavoured or plain yogurt (1 pot, 150 g) | 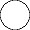 | 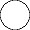 | 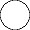 | 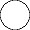 | 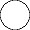 | 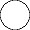 | 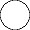 | 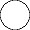 | 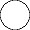 | 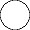 |
| Hard cheese – Gouda cheese, Edam cheese etc. (2 slices, 30 g) | 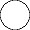 | 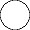 | 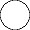 | 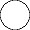 | 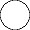 | 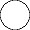 | 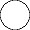 | 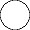 | 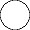 | 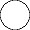 |
| Blue cheese (1 piece, 20 g) | 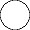 | 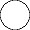 | 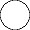 | 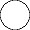 | 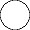 | 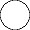 | 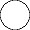 | 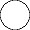 | 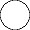 | 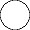 |
| Cottage cheese, Mozzarella, other types of processed cheese (3 tablespoons, 50 g) |  |  |  |  |  |  |  |  |  |  |
| Ice cream (1 scoop, 40 g) |  |  |  |  |  |  |  |  |  |  |
| Butter (1 teaspoon, 6 g) |  |  |  |  |  |  |  |  |  |  |
| Margarine (1 teaspoon, 6 g) |  |  |  |  |  |  |  |  |  |  |
| Eggs (1 egg, 50 g) |  |  |  |  |  |  |  |  |  |  |
| Egg pasta (100 g of dry pasta) |  |  |  |  |  |  |  |  |  |  |
| Red meat (steak in size of a hand, 100 g) |  |  |  |  |  |  |  |  |  |  |
| Poultry (steak in size of a hand, 100 g) |  |  |  |  |  |  |  |  |  |  |
| Meet products (3 thin slices of salami, 2 slices of Mortadella, 40 g) |  |  |  |  |  |  |  |  |  |  |
| Calf’s liver (1 cup, 60 g) |  |  |  |  |  |  |  |  |  |  |
| Mushrooms (6 pieces of common mushroom, 100 g) |  |  |  |  |  |  |  |  |  |  |
| Cakes, pastry and muffins (size of a muffin, 70g) |  |  |  |  |  |  |  |  |  |  |
